# Positive Relationship Between Precompetitive Sympathetic Predominance and Competitive Performance in Elite Extreme Sports Athletes

**DOI:** 10.3389/fspor.2021.712439

**Published:** 2021-08-23

**Authors:** Seiji Matsumura, Ken Watanabe, Naoki Saijo, Yuuki Ooishi, Toshitaka Kimura, Makio Kashino

**Affiliations:** NTT Communication Science Laboratories, Nippon Telegraph and Telephone Corporation, Atsugi, Japan

**Keywords:** autonomic nervous system activity, sympathetic predominance, heart rate variability, competitive performance, extreme sports, snowboarding

## Abstract

Elite athletes achieve superior performance under high pressure in competitive situations. Although it is known that such situations affect the precompetitive activity of their autonomic nervous system (ANS), the relationship between precompetitive ANS activity and performance remains controversial. Especially in extreme sports, it has been shown that cardiac sympathetic tone occurs in athletes before competition attempts. However, the relationship between precompetitive sympathetic tone and performance is unclear. To investigate this relationship in extreme sports, we organized a freestyle snowboard jumping competition and examined competitors' physiological states and performance during this event. The electrocardiograms (ECGs) of 20 elite snowboarders were measured 10 min before each jump in different competitive situations: *practice, qualifying*, and *final sessions*. The mean heart rate (HR), the low-frequency to high-frequency component ratio (LF/HF ratio), the logarithm of the HF (lnHF) component of the frequency-domain of the heart rate variability (HRV), the ratio of the standard deviation of all R–R intervals to the root mean square of successive differences of R–R intervals (SDNN/rMSSD ratio), and the rMSSD of the time-domain of the HRV were calculated from the ECG data. The results showed a significant increase in the mean HR as well as significant decreases in the lnHF component and rMSSD of the HRV as the *sessions* progressed. Interestingly, the mean HR, LF/HF ratio and SDNN/rMSSD ratio of the HRV showed significant positive correlations with competitive scores, and the lnHF component and rMSSD of the HRV showed significant negative correlations with the scores. Our results indicate that precompetitive ANS activity becomes predominantly sympathetic in elite extreme athletes, such as freestyle snowboarders, when the competition intensifies, and that this sympathetic predominance is positively related to competitive performance.

## Introduction

Elite athletes are able to achieve superior performance under high pressure in real-world game situations. Such situational characteristics affect their psychophysiological state (Mangine et al., 1990; Lazarus, 2000; Noblet and Gifford, 2002). Previous studies have reported that elite athletes demonstrate remarkable competitive performance, especially under the influence of changes in their precompetitive psychophysiological state (Smith et al., 1988; Sartor et al., 2013; Dalamitros et al., 2019).

The psychophysiological state during competition is reflected in the activity of the autonomic nervous system (ANS) (Aubert et al., 2003; Dong, 2016; Vitale et al., 2019). The ANS consists of the sympathetic nervous system (SNS) and the parasympathetic nervous system (PNS). It is well established that ANS activity can be estimated from physiological measurements (Malik, 1996). Several studies have examined how precompetitive ANS activity is related to the competitive performance in sports. For example, it has been reported that a decrease in the heart rate (HR) before a competitive attempt was associated with better competitive performance in shooting and archery, suggesting that the predominance of the PNS activity is advantageous for performance in these sports (Carrillo et al., 2011; Ortega and Wang, 2018). Another study had shown that golf putting performance decreased with an increase in HR and anxiety when the player was under pressure, suggesting that predominance of the SNS activity was detrimental to that performance (Weinberg and Genuchi, 1980). Such performance decay is known as “choking under pressure” (Baumeister, 1984; Nousiainen et al., 2001; Mateo et al., 2012; Schneider et al., 2018).

In contrast, the sympathetic tone has positive effects on motor output in certain situations. For example, in military parachute jumping, an increase in the HR before jumping was associated with improved motor performance after the jump, suggesting a positive effect of the increased SNS activity and the fight-or-flight response (Clemente-Suárez et al., 2017). It has also been shown that sympathetic nerve activity leads to acute proprioceptive sensitivity, which is important for coordinated whole-body movements (Matre and Knardahl, 2003; Horslen and Carpenter, 2011). However, there is little evidence regarding the positive effects of the SNS activity on athletes' performance in a real-world sporting situation.

One possible reason why the effect of the sympathetic tone on sports performance is still unclear is that the characteristics of sports have not been considered in such analysis. Performance in shooting and archery, which might be impaired by the excessive sympathetic tone, requires precision with minimal whole-body movement (Mitchell et al., 2005; Carrillo et al., 2011; Ortega and Wang, 2018). In contrast, SNS activity predominates in elite BMX competitors during their progression from practice to competition or while they wait at the beginning of a competition attempt (Mateo et al., 2012). Such sports are categorized as extreme sports and require instantaneous, intensive, and physically risky actions (Mitchell et al., 2005; Korobeynikov et al., 2016; Cohen et al., 2018). However, it is still unclear whether increased precompetitive sympathetic tone and performance are positively related.

The main purpose of this study was to investigate the effect of precompetitive sympathetic predominance on competitive performance in extreme sports. We focused on elite snowboarders in a freestyle snowboard jumping event (The International Snowboard Competition Rules, 2017). Because snowboarding is categorized as an extreme sport, similar to BMX, the precompetitive ANS activity is expected to exhibit sympathetic predominance as the competitive situation becomes more intense (Mateo et al., 2012; Korobeynikov et al., 2016; Cohen et al., 2018). Additionally, because snowboarders show a single trick during one attempt in the competition, which is scored by judges, it is possible to examine the relationship between the competitive performance and the physiological state before the attempt. We organized a snowboard jumping competition to control the experimental environment. The general format of this competition included *practice, qualifying*, and *final sessions*. We used electrocardiograms (ECGs) to estimate ANS activity 10 min before the jump in each *session*. First, we compared the indices of ANS activity between different *sessions*. We then investigated correlations between the indices of ANS activity before each jump and the competitive scores given by judges for the jump. We expected that snowboarders would exhibit sympathetic predominance before the jump attempt and that the degree of sympathetic predominance would be positively related to the subsequent competitive performance.

## Materials and Methods

### Participants

Twenty finalist snowboarders in the competition (19 males and one female, 22 ± 8 [mean ± SD] years) were included in this study. A total of 44 snowboarders took part in this competition and were subjected to physiological measurements in the *practice* and *qualifying sessions*. However, only 20 finalists in the *final session* provided additional physiological measurements from that *session*. We focused on the 20 finalists for the analysis because we sought to compare physiological states between all *sessions* in this competition. The finalists' skill level was advanced, and 12 of the 20 finalists were professional snowboarders (11 males and one female, length of the professional career: 3.3 ± 1.2 [mean ± SD] years). As this study involved human participants, it was reviewed and approved by the Ethics and Safety Committees of NTT Communication Science Laboratories (protocol H30-002). The participants or the participants' legal guardian/next of kin provided their written informed consent to participate in this study. They were also given the option to withdraw at any time without penalty. The study was conducted in accordance with the Declaration of Helsinki.

### Procedure

The snowboard jumping competition was held on a 5-meter jump slope on an indoor ski slope (SNOVA Shin-Yokohama, Japan). The temperature at the slope was −1.5 ± 1.5 °C. All measurements were performed on the same day. Experiments were conducted between 11:00 and 21:00 h.

This competition closely matched the characteristics of regular professional competitions and included *practice, qualifying*, and *final sessions*. All participants in the competition jumped twice in the *practice session* and twice in the *qualifying session*. Three judges scored the performance in each jump. The total score of the three judges determined the competitive ranking of the participants, and the top 20 participants in the *qualifying session* advanced to the *final session*. The highest-scoring participants in the *final session* received a prize (600,000 Japanese yen). The similarity to an actual competition event ensured that the athletes took their participation seriously and enabled us to examine physiological changes in a real competitive situation.

The schedule of this competition was as follows. Snowboarders arrived at the competition venue between 11:00 and 14:00 h and registered for participation. After registration, the participants were equipped with a wearable HR monitor and then warmed up. During the warm-up, participants could not jump but were allowed to stretch and snowboard on the slope. The *practice session* was held between 14:00 and 16:30 h, and all participants jumped twice. One participant dropped out from the competition because of an injury. The *qualifying session* was held between 16:30 and 19:00 h with 43 participants. The participants jumped twice in the same way as in the *practice session*. The *qualifying* scores were aggregated between 19:00 and 19:30 h, and the *qualifying* ranking was displayed. The top 20 participants in the *qualifying session* then proceeded to the *final session*. During this period, the participants took a break or warmed up. The *final session* was held between 19:30 and 21:00 h. The 20 finalists, who were included in the analysis, jumped two more times.

### Physiological Measurements

The ECG was measured for all participants during the competition. The measurements were recorded 10 min before the jump. For each measurement, data were recorded for 120 s while the participants were in a sitting position. After the measurement, participants prepared and waited for the start of the jump and then began the jump. During the physiological recordings, the participants were encouraged to relax. Participants were prohibited from drinking alcohol or smoking during the competition. From 10 min before the physiological measurements to the end of the corresponding jump, food or drink intake was restricted, and the participants were required to remain at rest without performing any exercise.

All participants were equipped with a wearable HR monitor (hitoe^®^, Toray Industries, Inc., Tokyo, Japan) to provide ECG data. The electrodes were constructed from hitoe^®^, which is an electrically conductive, hydrophilic fabric with good adhesion to the skin (Tsukada et al., 2012). The ECG data were obtained using a hitoe^®^ transmitter (NTT DOCOMO, Inc., Tokyo, Japan) and recorded at 200 Hz. The ECG data were then sent from the hitoe^®^ transmitter to a smartphone, which each participant carried. The wearable HR monitor hitoe^®^ is designed for use while performing sports activities (Higuchi et al., 2019).

### Performance Evaluation

Three judges, who were also professional snowboarders, scored each jump performance in terms of the *Amplitude, Difficulty, Execution, Progression*, and *Landing* (The International Snowboard Competition Rules, 2017). The scoring procedure was divided into two steps. In the first step, the judges evaluated the *Landing*. If the player fell completely, the score was 0. After evaluating the *Landing*, the judges provided scores related to other scored criteria only for successful landing attempts. The basal score was determined by the *Difficulty* (e.g., the number of spins). The *Amplitude, Execution*, and *Progression* were evaluated as additional scores. The basal score ranged from 0 to 100 points, and each additional score ranged from 0 to 10 points. Therefore, scores correlated with the *Difficulty*. The total score of the three judges determined the competitive ranking of the participants.

### Data Analysis

The performance parameters of the 20 finalists were analyzed. From the measurements obtained for these finalists, accurately recorded data of 15 finalists (14 males and one female) were used for the final analysis. Data from five finalists were excluded because of measurement errors (e.g., data transmission error and data loss due to short-circuiting between electrodes caused by excessive sweating or because electrodes did not make sufficiently tight contact with the skin).

The R–R intervals were obtained from the ECG measurements. To calculate the R–R intervals, the peak ECG signal was detected, and the result was visually screened to eliminate any inappropriate R-wave detection related to movement artifacts. The appropriately collected R–R interval data were resampled at 10 Hz using cubic spline interpolation. For the HR analysis, these resampled R–R intervals were converted into second-by-second values and expressed as beats per minute (BPM) by dividing 60 by each R–R interval value. Changes in the HR were evaluated by averaging the converted R–R intervals. The HR value averaged using this method was used as the mean HR for each condition. Because the mean HR is affected by the actions of both SNS and PNS on the cardiovascular system, it is an index of the relative relationship between sympathetic and parasympathetic cardiac modulations.

Heart rate variability (HRV) was then calculated to estimate the magnitude of ANS activity (Malik, 1996; Berntson et al., 1997; Dong, 2016). In HRV, both frequency-domain and time-domain analyses were conducted. In the frequency-domain analysis, discrete Fourier transformation (DFT) without zero padding was applied to the resampled R–R intervals. Prior to the DFT, the linear trend was removed, and a Hanning window was applied. The low-frequency (LF) component was obtained by integrating the power spectra over their respective ranges from 0.04 to 0.15 Hz, and the high-frequency (HF) component was obtained by integrating the power spectra over their respective ranges from 0.15 to 0.40 Hz. Subsequently, the LF to HF component ratio (LF/HF ratio), and the magnitude of the HF, using the natural logarithm of the HF power (lnHF), were evaluated. In the time-domain analysis, the standard deviation of all R–R intervals (SDNN) and the root mean square of successive differences of R–R interval (rMSSD) values were calculated. Subsequently, the SDNN to rMSSD ratio (SDNN/rMSSD ratio) was evaluated. The LF/HF ratio of the frequency-domain of the HRV and the SDNN/rMSSD ratio of the time-domain of the HRV are indices of the cardiac sympathovagal balance (Nousiainen et al., 2001; Kondo et al., 2002; Waring et al., 2008; Kang et al., 2016; Zajaczkowski et al., 2018). The lnHF component of the frequency-domain of the HRV and the rMSSD of the time-domain of the HRV are indices of the parasympathetic cardiac modulation (Larsen et al., 2010; Watanabe et al., 2015; Laborde et al., 2017).

First, the effects of the competitive *sessions* on each physiological index were examined. The mean HR, lnHF component, rMSSD, LF/HF ratio, and SDNN/rMSSD ratio of the HRV values were obtained for each participant in each *session*. The physiological values recorded for both jumps in each *session* were averaged for each participant. The difference in each physiological index between the competitive *sessions* was analyzed using one-way repeated-measures analysis of variance (ANOVA) with *session* as the “within” factor followed by the *post hoc* Holm's test.

The relationship between the precompetitive physiological state and competitive performance was examined. The physiological values were normalized by the average values for each participant in all *sessions* to allow comparisons. To compare the differences between judges' scores for the participants, we obtained the difference between the scores in each *session* and the average score for all *sessions* for each participant. We defined this difference as score variation. The Pearson's correlation test was used to examine the statistical correlation between the normalized physiological values and score variations. In this second analysis, completely failed jumps were eliminated because the score of these jumps was 0, without any consideration of other judging criteria, and these jumps could not be compared with successfully landed jumps. JASP (ver. 0.14.1) statistical software was used for all analyses. Statistical significance was set at *p* < 0.05.

## Results

### Changes in the Physiological State During Competitive *Sessions*

**Figure 1:**
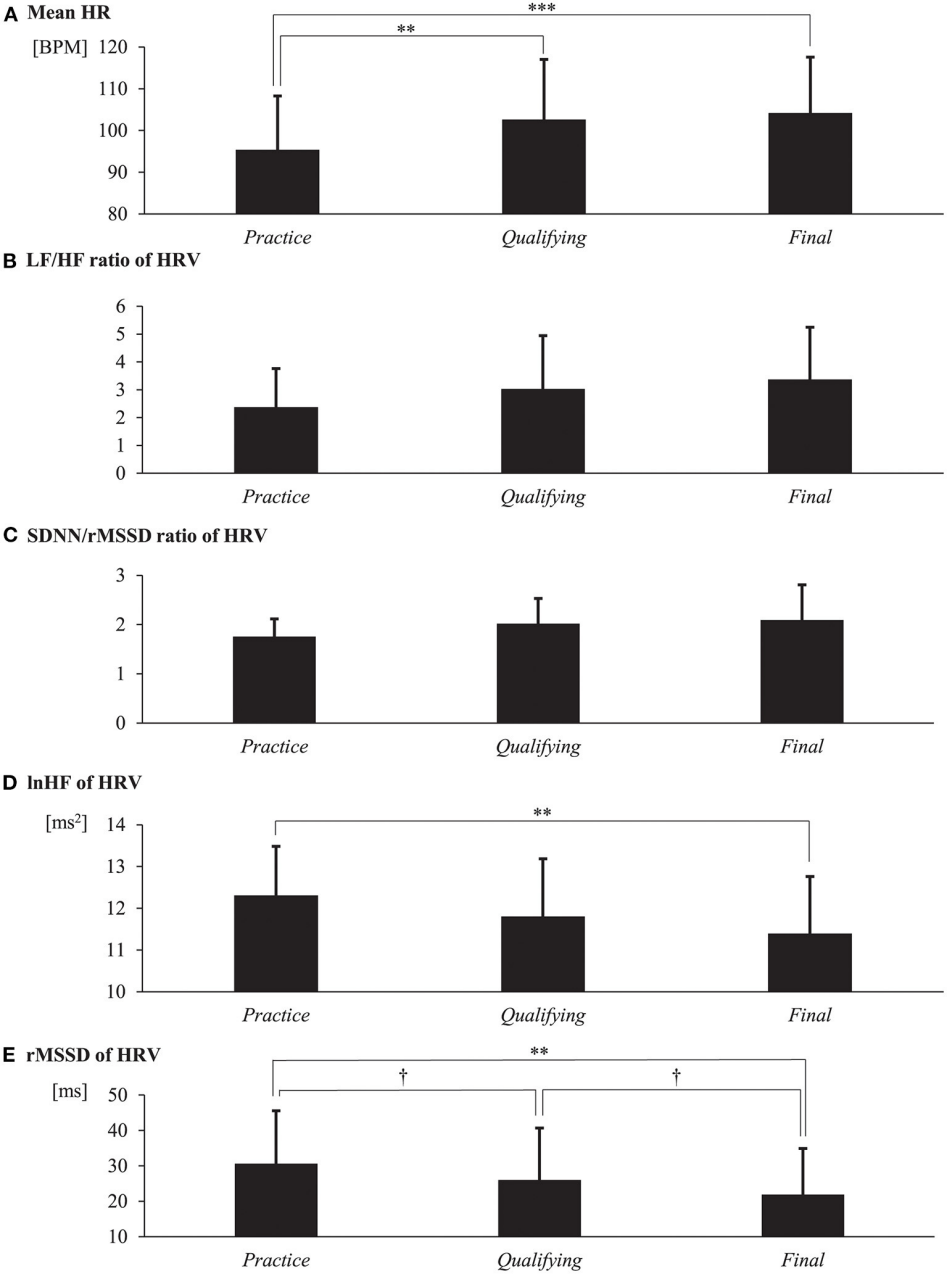
Physiological data obtained during different *sessions*. **(A)** Mean heart rate (HR). **(B)** The ratio of the low-frequency component to the high-frequency component (LF/HF ratio) of the heart rate variability (HRV). **(C)** The ratio of the standard deviation of all R–R intervals to the root mean square of successive differences of R–R intervals (SDNN/rMSSD ratio) of the HRV. **(D)** The logarithm of the HF (lnHF) component of the HRV. **(E)** The rMSSD of the HRV. Data are presented as the mean ± standard deviation. The Holm's method was employed for *post hoc* testing. Statistical significance of differences is indicated as follows: ^†^*p* < 0.1, **p < 0.01, ***p < 0.001.

To examine the effects of the characteristics of the competitive *sessions*, we compared the physiological indices during different *sessions*. Figure 1 shows the mean physiological indices of the participants.

Figure 1A shows the mean HR for each *session* across the subjects. The mean values and standard deviations (SD) were as follows: 95.420 ± 12.840 (*practice*), 102.652 ± 14.404 (*qualifying*), and 104.240 ± 13.311 (*final*). The mean HR was measured in BPM units. One-way repeated-measures ANOVA indicated a significant effect of *session* on the mean HR (*F*_(2, 28)_ = 10.226, *p* < 0.001, partial η^2^ = 0.422). The *post hoc* Holm's test revealed that the mean HR values in the *qualifying* and *final sessions* were significantly larger than those in the *practice session* (*practice*-*qualifying*: *t* = −3.478, *p* = 0.003, 95% CI = [−12.526, −1.937], Cohen's *d* = −0.898; *practice*-*final*: *t* = −4.242, *p* < 0.001, 95% CI = [−14.115, −3.526], Cohen's *d* = −1.095).

Figure 1B shows the mean LF/HF ratio of the HRV in each *session*. The mean and SD values were as follows: 2.381 ± 1.379 (*practice*), 3.029 ± 1.913 (*qualifying*), and 3.372 ± 1.878 (*final*). Although the LF/HF ratio of the HRV tended to increase as the *session* progressed, the one-way repeated-measures ANOVA indicated no significant effect of *session* (*F*_(2, 28)_ = 1.496, *p* = 0.241, partial η^2^ = 0.097).

Figure 1C shows the mean SDNN/rMSSD ratio of the HRV. The mean and SD values were as follows: 1.757 ± 0.357 (*practice*), 2.018 ± 0.510 (*qualifying*), and 2.095 ± 0.710 (*final*). Although the SDNN/rMSSD ratio of the HRV tended to increase as the *session* progressed, the one-way repeated-measures ANOVA indicated no significant effect of *session* (*F*_(2, 28)_ = 3.098, *p* = 0.061, partial η^2^ = 0.181).

Figure 1D shows the mean lnHF of the HRV. The mean and SD values were as follows: 12.313 ± 1.170 (*practice*), 11.804 ± 1.379 (*qualifying*), and 11.395 ± 1.362 (*final*). The lnHF of the HRV was measured in ms^2^ units. One-way repeated-measures ANOVA indicated a significant effect of *session* on the mean lnHF of the HRV (*F*_(2, 28)_ = 6.072, *p* = 0.006, partial η^2^ = 0.302). The *post hoc* Holm's test revealed that the lnHF of the HRV in the *final session* was significantly smaller than that in the *practice session* (*t* = 3.478, *p* = 0.005, 95% CI = [0.246, 1.591], Cohen's *d* = 0.898).

Figure 1E shows the mean rMSSD of the HRV. The mean and SD values were as follows: 30.659 ± 14.916 (*practice*), 26.009 ± 14.695 (*qualifying*), and 21.912 ± 12.991 (*final*). The rMSSD of the HRV was measured in ms units. One-way repeated-measures ANOVA indicated a significant effect of *session* on the mean rMSSD of the HRV (*F*_(2, 28)_ = 8.243, *p* = 0.002, partial η^2^ = 0.371). The *post hoc* Holm's test revealed that the rMSSD of the HRV in the *final session* was significantly smaller than that in the *practice session* (*t* = 4.058, *p* = 0.001, 95% CI = [3.258, 14.236], Cohen's *d* = 1.048). The *post hoc* test also revealed that the rMSSD of the HRV in the *qualifying session* tended to be smaller than that in the *practice session*, and in the *final session*, it tended to be smaller than that in the *qualifying session* (*practice*-*qualifying*: *t* = 4.650, *p* = 0.079, 95% CI = [−0.840, 10.139], Cohen's *d* = 0.557; *qualifying*-*final*: *t* = 4.097, *p* = 0.079, 95% CI = [−1.392, 9.587], Cohen's *d* = 0.491).

### Relationship Between Physiological State and Competitive Performance

**Figure 2:**
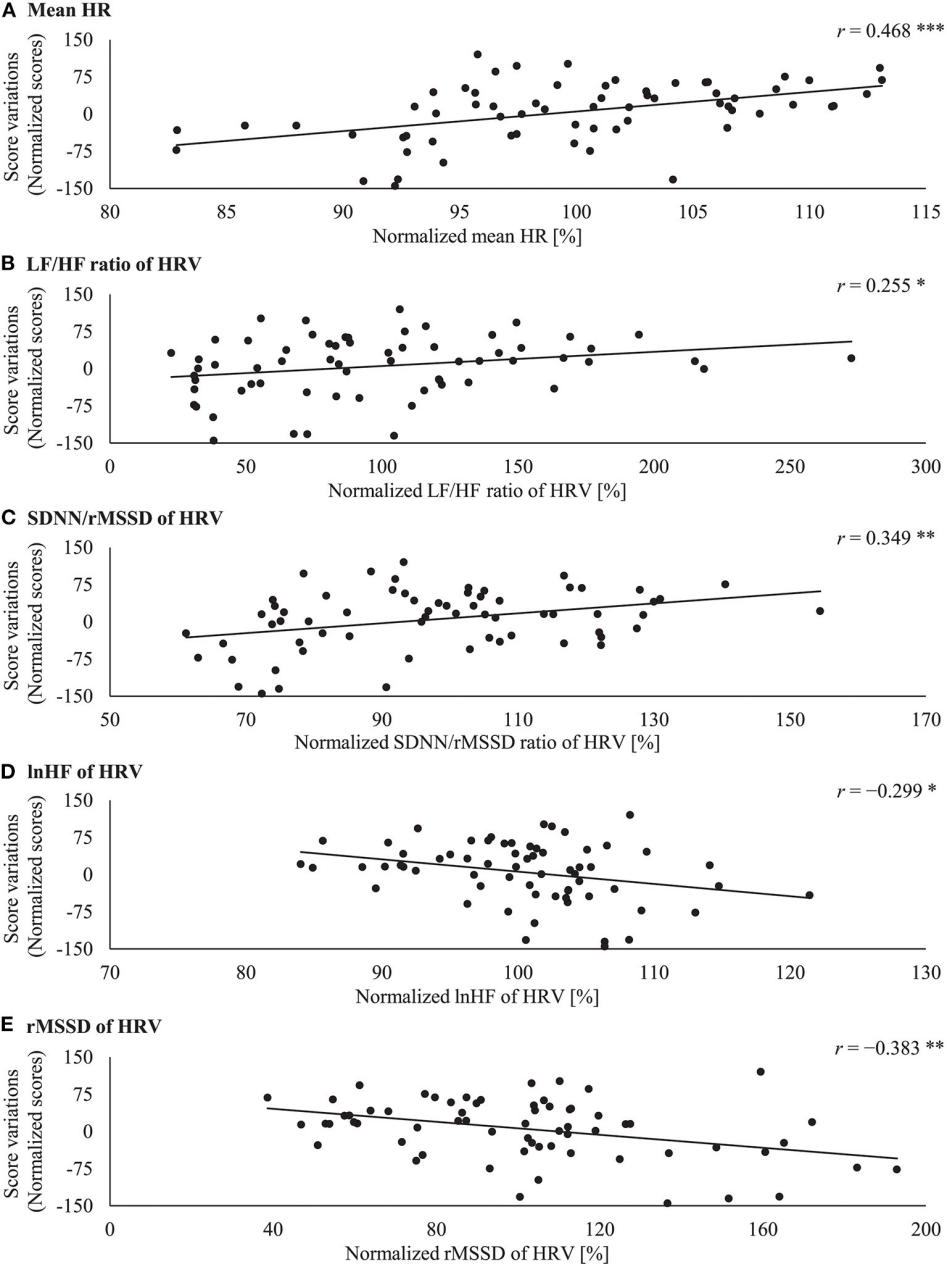
Correlations between normalized score variations and **(A)** mean HR, **(B)** LF/HF ratio of the HRV, **(C)** SDNN/rMSSD ratio of the HRV, **(D)** lnHF component of the HRV, and **(E)** rMSSD of the HRV. The vertical axis indicates score variations (the difference between the points scored in each of the three *sessions* and the average score for all *sessions* for each participant). The scores, including score variations, are given as points. The horizontal axis indicates the normalized physiological data (percentage of the average values for all *sessions* for each participant). The solid line indicates a linear regression line for the data. There are 65 data points in each graph. Statistical significance is indicated as follows: *p < 0.05, **p < 0.01, ***p < 0.001.

Figure 2 shows the relationship between the normalized physiological indices and score variation. Physiological indices of each participant were normalized by the average values of all *sessions*. The score variation was defined as the difference between the score in each *session* and the mean score for all *sessions* for each participant. The normalized mean HR and the normalized SDNN/rMSSD ratio of the HRV showed a significant positive moderate correlation with the score variation (mean HR: *N* = 65, *r* = 0.468, *p* < 0.001, 95% CI = [0.253, 0.639]; SDNN/rMSSD ratio of the HRV: *N* = 65, *r* = 0.349, *p* = 0.004, 95% CI = [0.115, 0.546]). The normalized LF/HF ratio of the HRV also showed a significant positive weak correlation with the score variation (*N* = 65, *r* = 0.255, *p* = 0.041, 95% CI = [0.011, 0.469]). In contrast, the normalized rMSSD of the HRV showed a significant negative moderate correlation with the score variation (*N* = 65, *r* = −0.383, *p* = 0.002, 95% CI = [−0.573, −0.153]). The normalized lnHF component of the HRV also showed a significant negative weak correlation with the score variation (*N* = 65, *r* = −0.299, *p* = 0.016, 95% CI = [−0.506, −0.059]).

## Discussion

### Precompetitive ANS Activity and Competitive Performance

In the current study, we investigated the relationship between the precompetitive physiological state and the competitive performance of freestyle snowboarders. The physiological state significantly changed during different *sessions* (Figure 1). When the competitive situation became more intense, such as when the participants progressed to the *final session*, the mean HR increased, and the lnHF component and rMSSD of the HRV decreased significantly. These physiological effects significantly correlated with differences in competitive performance (Figure 2). The mean HR, and LF/HF and SDNN/rMSSD ratio of the HRV positively correlated with the competitive scores. Conversely, the lnHF and rMSSD of the HRV negatively correlated with the competitive scores.

Regarding the effect of the competitive situation on the ANS activity, the current results revealed that the PNS activity of freestyle snowboarders decreased when the *session* progressed from *practice* to *final*. The lnHF component and rMSSD of the HRV (indices of the parasympathetic cardiac modulation) were significantly lower in the *final session* than in the *practice session*. In addition, the mean HR values in the *qualifying* and *final sessions* were significantly higher than those in the *practice session*, and the LF/HF and SDNN/rMSSD ratio of the HRV (indices of the cardiac sympathovagal balance) tended to increase as the *session* progressed. Given the previous finding that the PNS and SNS are activated inversely (McKitrick and Calaresu, 1996), the current results indicate that the precompetitive ANS activity of freestyle snowboarders becomes predominantly sympathetic when the competitive *session* becomes more intense. This sympathetic predominance could be induced by psychological factors, such as the anticipation of victory and anxiety regarding the challenges involved, which competitors may experience more strongly as the competition becomes more intense (Rakover and Levita, 1973; Meyer et al., 2000; Blásquez et al., 2009; Mateo et al., 2012; Schneider et al., 2018). In general, the mean HR in a daily non-competitive resting state is approximately 60–80 BPM (Mason et al., 2007), and it increases by several BPM from the resting state due to the sympathetic tone in non-competitive indoor laboratory environments (Watanabe et al., 2015). On the other hand, in a real-world sporting game, where intense psychological pressure is experienced, it is known that the HR in a seated position before the competitive attempt increases to approximately 90–100 BPM (Mateo et al., 2012; Ortega and Wang, 2018). The HR observed in our study was in the range observed in other real-world sports studies; therefore, it may be concluded that our experimental design simulated a real-world competitive sporting situation.

Regarding the relationship between the precompetitive ANS activity and competitive performance, the mean HR, LF/HF, and SDNN/rMSSD values positively moderately or weakly correlated with the performance scores (Figures 2A–C). Additionally, the lnHF component and rMSSD of the HRV values negatively moderately or weakly correlated with the scores (Figures 2D,E). These results support our expectation that the precompetitive sympathetic predominance positively correlates with the competitive performance. Notably, in our study, the performance evaluation was a subjective assessment by judges, the amount of the data obtained from each individual was small, and the data of all subjects were integrated. These circumstances may have led to moderate or weak strengths of the correlations. Contrary to the results from previous studies involving archery and shooting (Carrillo et al., 2011; Ortega and Wang, 2018), our present results suggest that the sympathetic predominance of elite snowboarders improves competitive performance. This difference may depend on the characteristics of the required performance (Mitchell et al., 2005; Cohen et al., 2018). For example, in shooting or archery, static and precise control of movement are required to make an accurate shot. In contrast, a snowboard jump requires high exercise intensity and fluent coordination of the entire body in the air and involves physical risks. These differences may lead to different requirements for the precompetitive ANS activity.

There are at least two possible explanations for the correlation between sympathetic predominance and better performance. Because snowboard jumping events require full-body exercise and the parameters evaluated include the height of the jump and fluidity of the movements, both simple power output, and whole-body coordination are required for jumping attempts. One possibility is that the sympathetic predominance of snowboarders contributes to increased exercise intensity, enabling participants to jump higher. Previous studies have reported that automatic parachute jumping involves increased lower limb muscle strength due to sympathetic predominance (Clemente-Suárez et al., 2017), and elite rowing athletes become SNS predominant during very high-intensity training (Iellamo et al., 2002). Another possibility is that sympathetic predominance contributes to the sophisticated coordination of whole-body movement in the air. Proprioception is critical for inter-joint coordination (Sainburg et al., 1993), and proprioceptive sensitivity is positively related to SNS activity (Matre and Knardahl, 2003; Hjortskov et al., 2005; Kamibayashi et al., 2009). In snowboarders, high proprioceptive sensitivity might contribute to the ability to perform highly coordinated whole-body movements in the air. Further studies will be needed to investigate these possibilities, including testing when ANS activity improves motor output and whole-body coordination.

### Practical Applications

As mentioned earlier, the appropriate level of the precompetitive ANS activity for maximizing performance would be expected to differ between various sports. Our study is novel as it suggests that sympathetic predominance would be advantageous for performance in instantaneous, intensive, and physically risky sports such as snowboard jumping, which we tested by organizing an actual competitive event. From a practical perspective, our findings will be useful as a guideline, especially for extreme sports athletes, which would allow them to determine what their psychophysiological state should be just before the trial. In the future, the current findings also should be optimized for individual athletes by accumulating more intra-individual data. Moreover, our findings may also contribute to the development of appropriate and efficient methods for modulating precompetitive ANS activity.

### Limitations

This study had several limitations related to the measurement being performed in a real competitive situation. Several other possible factors could have affected the precompetitive psychophysiological state and performance because we focused at preserving the real competitive situation in this study. Although it is difficult to cover all other factors, some of them are discussed below.

In the context of sports, physiological characteristics are reportedly different between the sexes (Berkoff et al., 2007; Dong, 2016). To ensure a sufficient number of participants in our study, we adopted a free-entry system in the competition and did not control for sex. However, the results of an additional analysis, in which the participants were divided into males and females, showed the same trend as our original results with participants of both sexes combined (see Supplementary Figures 1 and 2), suggesting that the present findings would be significant in the context of elite extreme sports athletes irrespective of sex.

Given that our experiment was conducted in the later part of the day, diurnal variation needs to be considered. Previous studies have reported that HR decreases and becomes parasympathetic predominant from afternoon to night (Bondanelli et al., 1999; Kondo et al., 2002; Vandewalle et al., 2007). However, in our study, as the *session* progressed (from afternoon to night), the participants' HR increased, indicating sympathetic predominance. Because this physiological change was in the opposite direction compared to the expected circadian effect, we can assume that sympathetic predominance had the main influence on performance in the competitive situation in our setting.

Furthermore, the previous trials might have had an impact on the current precompetitive physiological state. Fatigue is one such specific factor. In previous studies, fatigue affected the physiological state and led to a decrease in performance (Schmitt et al., 2013, 2015). In this study, we tried to eliminate the influence of fatigue as much as possible by having an interval of at least 30 min between trials and ensuring that the participants rested for 10 min before the trial. Furthermore, if the physiological state changed due to the influence of fatigue, the performance would have decreased. However, our results showed that performance improved as the *session* progressed, despite accumulating fatigue, suggesting that the latter factor did not have a major influence on the physiological state during the competitive situation.

Potential effects of water and food intake were minimized by the requirement to refrain from consuming water or food from 10 min before the trial until the end of that trial, however, we did not control the athletes during the rest of the period, respecting the real competitive settings. Reportedly, water and food intake can affect ANS activity for hours (Nacht et al., 1987; Christiani et al., 2021), and we can hardly claim that the influence of water and food intake was completely eliminated in this study.

As mentioned above, several limitations should be considered due to the acquisition of real-world measurements. However, because at least the competitive situation (*session*) was well controlled in our study, we can still claim that the ANS activity becomes sympathetic predominant as the competitive situation becomes more intense (as the *session* progressed), and this predominance is positively related to the performance.

## Conclusions

In the current study, we investigated the effects of different precompetitive situations on the ANS activity and examined the relationship between the precompetitive ANS activity and competitive performance of elite extreme sports athletes in a real-world competition. Our results indicate that precompetitive ANS activity of elite extreme sports athletes becomes predominantly sympathetic when the competitive situation becomes more intense, and that their sympathetic predominance is positively related to competitive performance.

## Data Availability Statement

The raw data supporting the conclusions of this article will be made available by the authors, without undue reservation.

## Ethics Statement

The studies involving human participants were reviewed and approved by The Ethics and Safety Committees of NTT Communication Science Laboratories. Written informed consent to participate in this study was provided by the participants or the participants' legal guardian/next of kin.

## Author Contributions

SM, KW, and MK: conceptualization. SM, KW, NS, TK, and MK: methodology. SM and KW: software. SM, KW, NS, YO, TK, and MK: formal analysis, validation and writing—review and editing. SM, KW, and NS: investigation. SM, KW: resources, data curation, project administration, and visualization. SM: writing—original draft preparation. MK: supervision and funding acquisition. All authors have read and agreed to the published version of the manuscript.

## Conflict of Interest

All the authors are employees of NTT Communication Science Laboratories, which is a basic-science research section of Nippon Telegraph and Telephone Corporation (NTT). There is a pending patent involving the reported research. There are no products in development or marketed products to declare. The pending patent does not alter the author's adherence to policies of Frontiers.

## Publisher's Note

All claims expressed in this article are solely those of the authors and do not necessarily represent those of their affiliated organizations, or those of the publisher, the editors and the reviewers. Any product that may be evaluated in this article, or claim that may be made by its manufacturer, is not guaranteed or endorsed by the publisher.
