# Supplementary material for: Positive Relationship Between Precompetitive Sympathetic Predominance and Competitive Performance in Elite Extreme Sports Athletes
Source: Front Sports Act Living. 2021 Aug 23;3:712439. doi: 10.3389/fspor.2021.712439 (PMC8421024; doi:10.3389/fspor.2021.712439)
Supplement: Supplementary file 1 [file Data_Sheet_1.PDF]

Supplementary Material

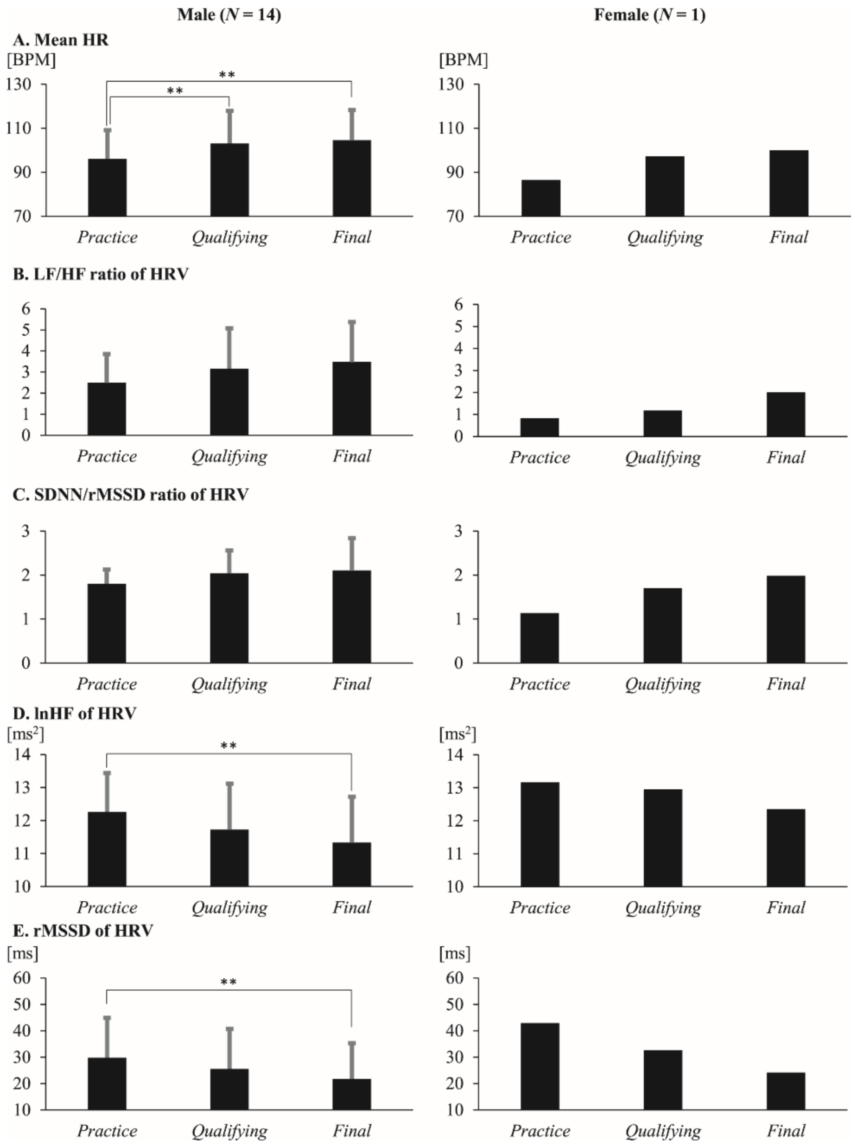

Supplementary Figure 1. Physiological data for each male and female obtained during different *sessions*. The left figures represent the results of males ($N = 14$), and the right figures represent the results of a female ($N = 1$). (A) Mean heart rate (HR). (B) The ratio of the low-frequency component to the high-frequency component (LF/HF ratio) of the heart rate variability (HRV). (C) The ratio of the standard deviation of all R-R intervals to the root mean square of successive differences of R-R intervals (SDNN/rMSSD ratio) of the HRV. (D) The logarithm of the HF (lnHF) component of the HRV. (E) The rMSSD of the HRV. Data are presented as the mean in the results of both sexes, and standard deviations are additionally used in the results of males. The Holm's method was employed for *post hoc* testing in the analysis of males. Statistical significance of differences is indicated as follows: $^{**}p < 0.01$.

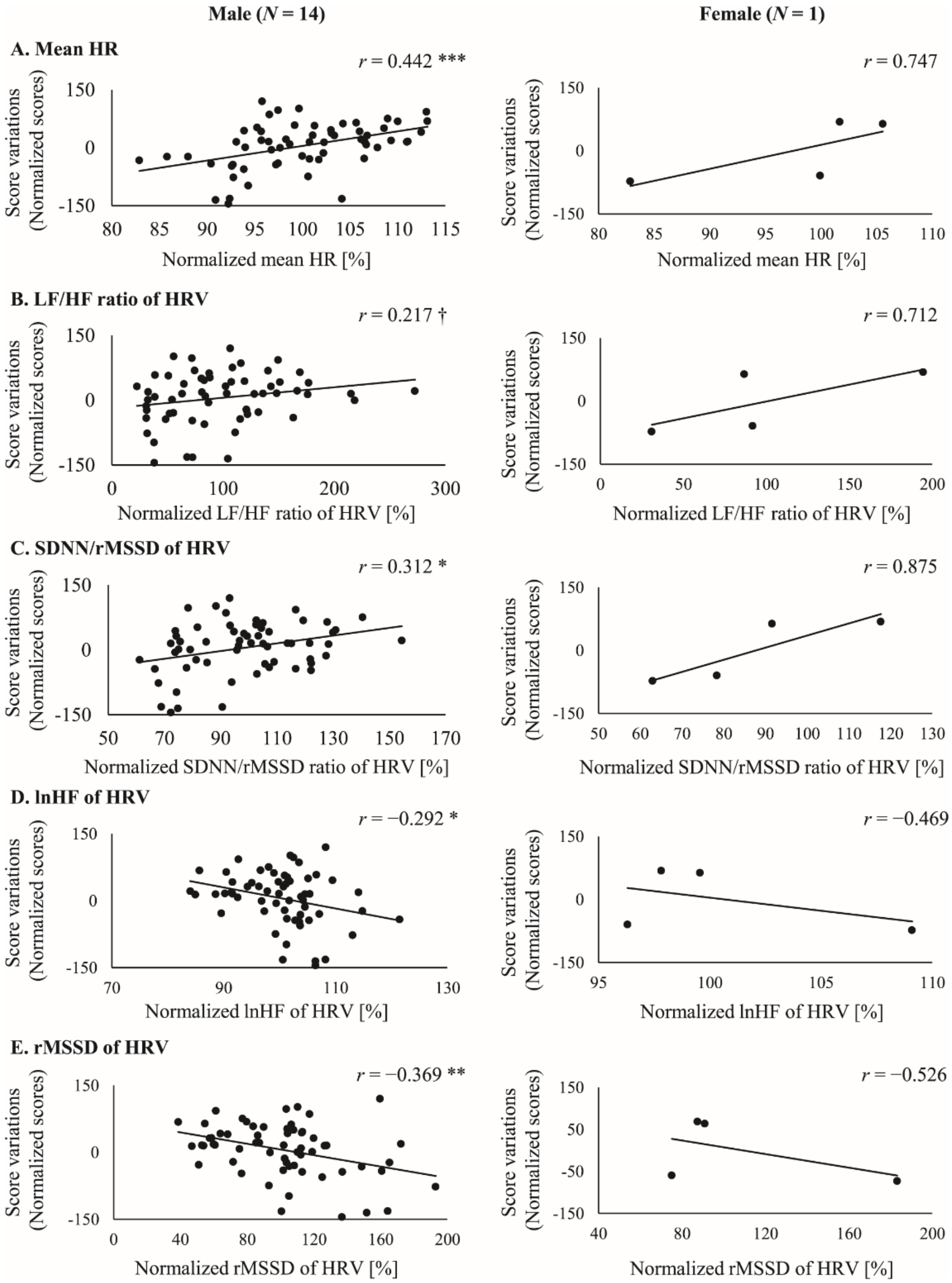

Supplementary Figure 2. Correlations for each male and female between normalized score variations and (A) Mean HR, (B) LF/HF ratio of the HRV, (C) SDNN/rMSSD ratio of the HRV, (D) lnHF component of the HRV, and (E) rMSSD of the HRV. The figures on the left represent the results of males ($N = 14$), and the figures on the right represent the results of a female ($N = 1$). The vertical axis indicates score variations (the difference between the points scored in each of the three sessions and the average score for all sessions for each participant). The scores, including score variations, are given as points. The horizontal axis indicates the normalized physiological data (percentage of the average values for all sessions for each participant). The solid line indicates a linear regression line for the data. There are 61 data points in graphs describing males, and there are only four data points in graphs describing the data from the only female. Statistical significance is indicated as follows: †$p < 0.1$, *$p < 0.05$, **$p < 0.01$, ***$p < 0.001$.
